# Pandemic HIV-1 Vpu overcomes intrinsic herd immunity mediated by tetherin

**DOI:** 10.1038/srep12256

**Published:** 2015-07-17

**Authors:** Shingo Iwami, Kei Sato, Satoru Morita, Hisashi Inaba, Tomoko Kobayashi, Junko S. Takeuchi, Yuichi Kimura, Naoko Misawa, Fengrong Ren, Yoh Iwasa, Kazuyuki Aihara, Yoshio Koyanagi

**Affiliations:** 1 Mathematical Biology Laboratory, Department of Biology, Faculty of Sciences, Kyushu University, Fukuoka, Fukuoka 8128581, Japan; 2 PRESTO, JST, Kawaguchi, Saitama 3320012, Japan; 3 CREST, JST, Kawaguchi, Saitama 3320012, Japan; 4 Laboratory of Viral Pathogenesis, Institute for Virus Research, Kyoto University, Kyoto, Kyoto 6068507, Japan; 5 Department of Mathematical and Systems Engineering, Shizuoka University, Hamamatsu, Shizuoka 4328561, Japan; 6 Graduate School of Mathematical Sciences, The University of Tokyo, Meguro-ku, Tokyo 1538914, Japan; 7 Laboratory for Animal Health, Department of Animal Science, Faculty of Agriculture, Tokyo University of Agriculture, Atsugi, Kanagawa 2430034, Japan; 8 Department of Bioinformatics, Medical Research Institute, Tokyo Medical and Dental University, Tokyo 1138510, Japan; 9 Institute of Industrial Science, The University of Tokyo, Meguro-ku, Tokyo 1538505, Japan; 10 Graduate School of Information Science and Technology, The University of Tokyo, Meguro-ku, Tokyo 1138656, Japan

## Abstract

Among the four groups of HIV-1 (M, N, O, and P), HIV-1M alone is pandemic and has rapidly expanded across the world. However, why HIV-1M has caused a devastating pandemic while the other groups remain contained is unclear. Interestingly, only HIV-1M Vpu, a viral protein, can robustly counteract human tetherin, which tethers budding virions. Therefore, we hypothesize that this property of HIV-1M Vpu facilitates human-to-human viral transmission. Adopting a multilayered experimental-mathematical approach, we demonstrate that HIV-1M Vpu confers a 2.38-fold increase in the prevalence of HIV-1 transmission. When Vpu activity is lost, protected human populations emerge (i.e., intrinsic herd immunity develops) through the anti-viral effect of tetherin. We also reveal that all Vpus of transmitted/founder HIV-1M viruses maintain anti-tetherin activity. These findings indicate that tetherin plays the role of a host restriction factor, providing ‘intrinsic herd immunity’, whereas Vpu has evolved in HIV-1M as a tetherin antagonist.

Human immunodeficiency virus type 1 (HIV-1), the causative agent of acquired immunodeficiency syndrome (AIDS), emerged around 100 years ago^1^ through zoonotic transmission of simian immunodeficiency viruses (SIVs) in chimpanzees (SIVcpz)^2^,^3^ and gorillas (SIVgor)^4^. According to molecular phylogenetic analyses, zoonotic transmission has occurred at least 4 times, leading to diversification of the virus into 4 different groups; namely, HIV-1 M (“major” or “main”), N (“new” or “non-M-non-O”), O (“outlier”), and P^5^. Among these 4 HIV-1 groups, HIV-1M alone is pandemic and currently infects more than 30 million people worldwide. However, why HIV-1M rapidly created a worldwide pandemic while the other groups remained endemic is unclear.

In 2009, Neil *et al*.^6^ and Van Damme *et al*.^7^ identified a cellular anti-HIV-1 protein called tetherin (also known as bone marrow stromal antigen 2 [BST2], CD317, and HM1.24). Tetherin impairs viral release by tethering bud virions to the surfaces of HIV-1-producing cells. Conversely, tetherin is antagonized by viral protein U (Vpu), an accessory protein encoded by HIV-1^6^,^7^. In addition, Vpu potently induces the degradation of CD4 molecules in infected cells^8^. Interestingly, HIV-1M Vpu alone possesses both of these abilities, while the Vpus of the other HIV-1 groups (i.e., N, O, and P) possess neither or only one ability^9^,^10^,^11^. Regarding anti-tetherin activity, HIV-1N Vpu is a much weaker tetherin antagonist than HIV-1M Vpu^11^,^12^, and the Vpus of HIV-1O and HIV-1P exert no adverse effect on tetherin^10^,^11^. From these observations, Kirchhoff proposed that the ability of Vpu to counteract tetherin has promoted human-to-human viral transmission^13^. However, because human-to-human spread cannot be experimentally replicated, this hypothesis remains speculative. Therefore, we conducted an experimental-mathematical investigation, and concluded that human tetherin is a potent inducer of intrinsic herd immunity in humans and that HIV-1M Vpu has acquired the ability to overcome this hurdle.

## Methods

### A structured epidemiological model based on the distribution of set-point viral loads

**Figure 1:**
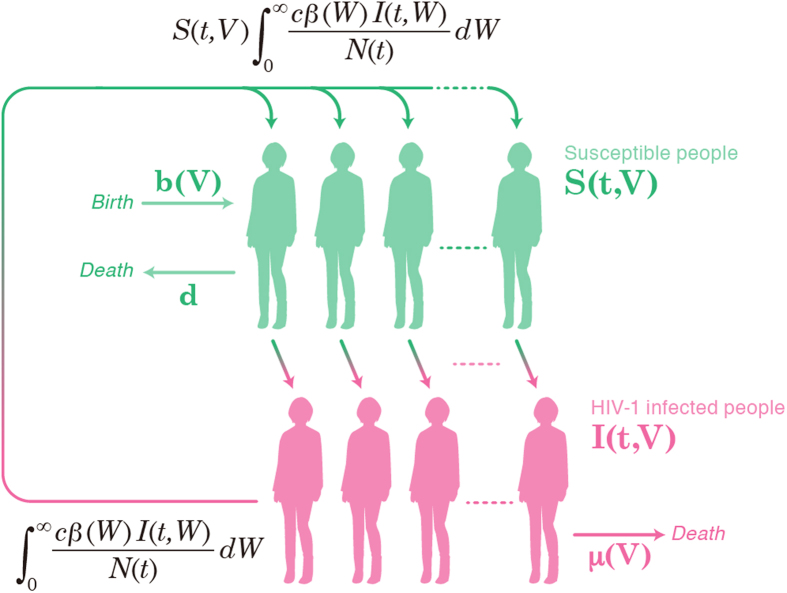
Construct of the mathematical model. The variables *S*(*t*, *V*) and *I*(*t*, *V*) denote the number of susceptible and infected individuals, respectively, and *N*(*t*) is the total number of host individuals. All variables are specified at time *t*. The parameters *b*(*V*) and *d* denote the birth and removal rates of susceptible individuals respectively, and *μ*(*V*) is the death rate of infected individuals. The force of infection is assumed as 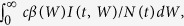 where *c* and *β*(*V*) denote the partner exchange rate and the transmission probability, respectively. Therefore, the rate of change in the number of susceptible and infected individuals at time *t* (i.e., the *de novo* transmission) is 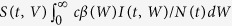. Figure 1 was drawn by KS using Illustrator CS5.1 (Adobe).

To investigate the relationship between the distributed set-point viral loads in HIV-1 infected patients and human-to-human viral transmission, we developed a novel mathematical model that accounts for host heterogeneity (i.e., the different set-point viral loads). The novel mathematical model is given by (Fig. 1):


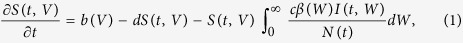



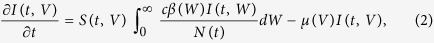


where *S*(*t*, *V*) represents the number of susceptible individuals who will acquire the set-point viral load *V* if infected by HIV-1 in the future, and *I*(*t*, *V*) is the number of infected individuals with the set-point viral load *V* at time *t*. *N*(*t*) is the total host population size (i.e., 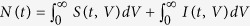). Here we assume that the set-point viral load *V* of each infected individual is determined by host properties such as the genetic background and immune state^14^,^15^,^16^,^17^. Host populations are born at rate *b*(*V*) and removed at rate *d*. Because most of the HIV infection occurs during the asymptomatic period and the transmission probability per partnership is correlated with the patients’ set-point viral load *V*, we model this relationship by an increasing Hill function as described elsewhere^18^,^19^; That is we write 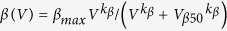 where *β*_*max*_ is the maximum transmission probability and *V*_*β*50_ is the viral load at which the probability is half its maximum. The parameter *k*_*β*_ is the steepness of the transmission probability versus the set-point viral load. Therefore, the force of infection at time *t* is given by 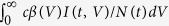 where *c* is the contact rate. Finally, we assume that the asymptomatic period endures as a decreasing Hill function of the viral load as described elsewhere^18^,^19^; that is, 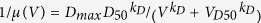 where *D*_*max*_ is the maximum duration of the asymptomatic period in years, *V*_*D*50_ is the viral load at which the duration is half its maximum, and *k*_*D*_ is the steepness of the (decreasing) duration versus the set-point viral load. The initial condition is *S*(0,*V*) = *b*(*V*)/*d* and 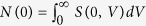. Similar mathematical models have been proposed in previous studies^18^,^19^,^20^.

### Cell culture, infection, transfection, Western blotting, TZM-bl assay, flow cytometry

293T cells, HeLa cells and TZM-bl cells (obtained through NIH AIDS Research and Reference Reagent Program) were maintained in DMEM (Sigma) medium containing FCS and antibiotics. Human PBMCs were maintained in RPMI1640 (Sigma) medium containing FCS and antibiotics. For *in vitro* HIV-1 infection assay (Figure S1), PHA-activated human PBMCs were infected with WT or vpu-deficient HIV-1 (strain NL4-3) at multiplicity of infection 0.1 (i.e., 10,000 TCID50 [50% tissue culture infectious dose] of virus solution was inoculated into 100,000 PHA-activated human PBMCs). The expression plasmids of HA-tagged Vpus of T/F viruses were obtained from GeneArt Gene Synthesis service (Life Technologies). Transfection was performed using Lipofectamine 2000 (Life Technologies). Western blotting, TZM-bl assay, and flow cytometry were performed as previously described^21^,^22^,^23^.

## Results

### HIV-1M Vpu increases the set-point viral load of HIV-1-infected humans

To examine whether or not the anti-tetherin ability of Vpu is linked to effective human-to-human HIV-1 transmission^13^, we performed a virus replication assay using primary human CD4^+^ T lymphocytes. The experimental data were analyzed by a previously proposed mathematical model^24^,^25^, which verified that Vpu increases the virus production rate (*p*) (see **Text S1**). We also analyzed 15 datasets provided in previous studies^7^,^26^,^27^,^28^,^29^,^30^,^31^ and found that the average production rate of wild-type HIV-1 was 1.54-fold higher than that of *vpu*-deficient HIV-1. The estimated parameter values are listed in Table S1. Because the virus production rate is known to affect the set-point viral load (*V*) in infected individuals^32^,^33^, we expected that a typical set-point of 1.0 × 10^5^ copies/ml (for example) would decrease to 0.4 × 10^5^ copies/ml in HIV-1M lacking *vpu* (see **Text S2**). These findings strongly suggest an important connection between Vpu and the within-host dynamics of HIV-1.

### Epidemiological impact of set-point viral loads

Because HIV-1-infected patients with higher viral loads are more infectious^34^,^35^ and have reduced lifespans^34^,^36^,^37^, the efficacy of human-to-human HIV-1 transmission is determined by the patients’ set-point viral load. In addition, the distribution of the set-point plays a critical role in viral spread^35^. We describe the human-to-human spread of HIV-1M by the structured epidemiological model described in **Methods** (Fig. 1). This model includes the transmission rate (*β*(*V*)) and the death rate of the patient (*μ*(*V*)) as functions of viral load^34^. Because patients’ set-point values are highly variable^34^,^35^, by adopting the set-point distribution approach in our mathematical model we can quantitatively detail the spread of HIV-1. Here we used the distribution of set-point viral loads in 311 untreated heterosexual HIV-1M infected patients in the Zambian transmission study^35^ and calculated the basic reproduction number (*R*_0_^*W*^), the expected number of people infected throughout their infectious lifespan^38^,^39^ (see **Text S3**). The estimated value of *R*_0_^*W*^ (4.67;see **Text S4**) is consistent with previous estimates^39^,^40^.

### Tetherin-mediated intrinsic herd immunity and its counteraction by Vpu

**Figure 2:**
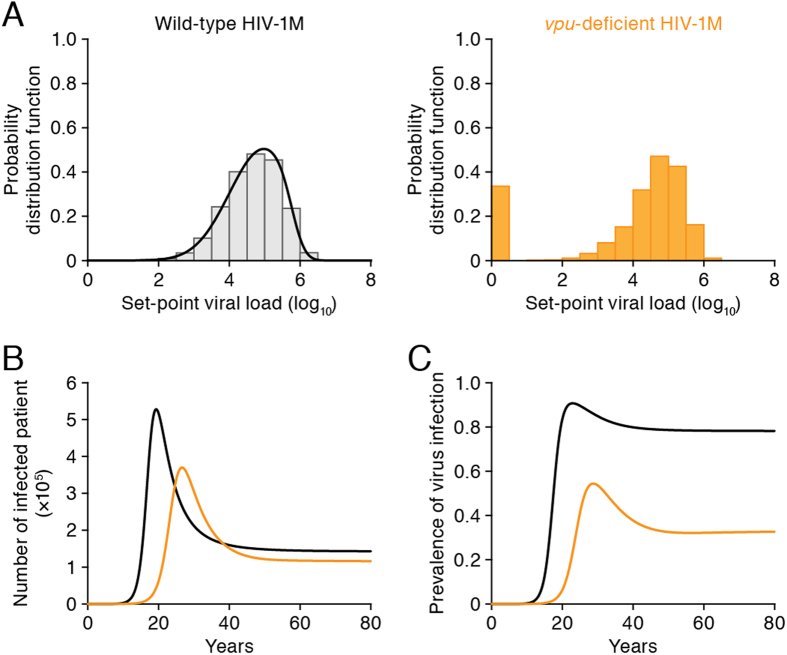
Prediction of tetherin-mediated intrinsic herd immunity. **(A)** (left) The distribution of set-point viral load in untreated individuals in the Zambian Transmission Study (black bars). The black curve is the distribution predicted by the structured epidemiological model. (right) The predicted distribution of set-point viral load in untreated individuals in the Zambian Transmission Study, assuming non-functional Vpu (orange bars). The bar at “0” shows the fraction of populations protected from *vpu*-deficient HIV-1 infection. **(B)** Simulated spread of wild-type and *vpu*-deficient HIV-1 among a population of 1 million individuals. The black and orange curves describe the number of individuals infected by wild-type and mutant virus, respectively. **(C)** Prevalence of wild-type (black curve) and *vpu*-deficient (orange curve) HIV-1 infection in (B).

To investigate the impact of Vpu on the human-to-human spread of HIV-1M, we assumed that HIV-1 has lost its Vpu function (i.e., we consider *vpu*-deficient HIV-1) and compared several quantities concerning the epidemiological contribution of Vpu. As explained above, viruses lacking Vpu reproduce more slowly and yield a lower set-point viral load in patients (see **Text S2**). Interestingly, *vpu*-deficient HIV-1 did not establish infection in some patients with lower set-point viral loads (<3.19 × 10^4^ RNA copies/ml) (see **Text S5**). In other words, the anti-viral effect of tetherin confers potential protection against HIV-1 infection among some human populations^41^. Because the distribution of viral loads in the Zambian transmission study is well described by a weighted skew-normal distribution of the logarithm of the viral load (see **Text S4**), we can estimate that 20% of human populations are protected from *vpu*-deficient HIV-1 by tetherin (Fig. 2A) and are prevented from transmitting HIV-1 (this situation is known as herd immunity). In addition, several observed distributions of the set-point are negatively skewed^34^,^35^, suggesting that tetherin-mediated herd immunity plays a critical role in the human-to-human spread of HIV-1M.

In *vpu*-deficient HIV-1, the calculated *R*_0_^*M*^ reduced to 3.90, suggesting that human-to-human transmission is 1.17-fold more efficient in wild-type HIV-1 than in *vpu*-deficient HIV-1. However, comparing the basic reproduction numbers may be of limited applicability when infected individuals are rare in a population. To further assess the impact of Vpu on the spread of HIV-1M, we simulated wild-type and *vpu*-deficient HIV-1 transmission among 1 million individuals (Fig. 2B). The parameter values used in these simulations are summarized in Table S2. Our simulations show that Vpu shortens the time between initial and peak HIV-1 infection and increases the steady-state number of infected individuals (Fig. 2B). Interestingly, the prevalence (i.e., the number of infected individuals divided by the number of total individuals) of wild-type and *vpu*-deficient HIV-1 infection were calculated as 78.2% and 32.8%, respectively (Fig. 2C). Thus, the Vpu of HIV-1M increases the viral prevalence by 2.38-fold, indicating that Vpu effectively overcomes tetherin mediated herd immunity.

### Conserved anti-tetherin ability of Vpu in transmitted/founder (T/F) viruses

**Figure 3:**
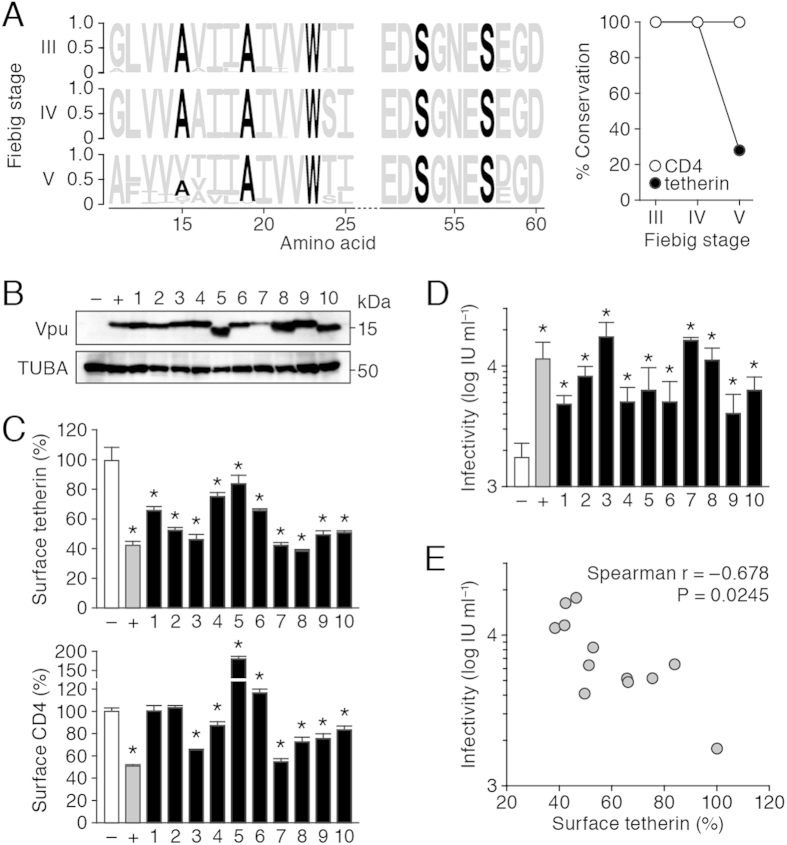
Conservation of Vpu motifs and the ability of T/F virus Vpus. **(A)** Conservation of the motifs in HIV-1M Vpus that counteract tetherin and down-regulate CD4 at each stage of viral infection. (left) Logo plot of the Vpu amino acid sequences at Fiebig stages III (n = 183), IV (n = 29), and V (n = 119). The crucial Vpu motifs for tetherin counteraction (AxxxAxxxA motif) and CD4 down-regulation (SxxxS motif) are indicated in black. (right) The percentage conservation of these motifs at each Fiebig stage. (**B**–**E**) Infection ability of the Vpus T/F viruses. The HA-tagged Vpu-expressing plasmids were co-transfected with pNL4-3*ΔvpuΔnef* (B, D, E) or without pNL4-3*ΔvpuΔnef* (**C**) into HeLa cells (B, D, E) or TZM-bl cells (C). Labels denote: –, empty vector; +, NL4-3 Vpu; 1-10, T/F virus Vpus: 1, THRO_TF1 (Genbank accession no. JN944930); 2, SUMA_TF1, (JN944928); 3, RHPA_TF1 (JN944917); 4, TRJO_TF1 (JN944936); 5, CH040_TF1 (JN944905); 6, REJO_TF1 (JN944911); 7, CH077_TF1 (JN944909); 8, CH058_TF1 (JN944907); 9, WITO_TF1 (JN944938); 10, CH106_TF1 (JN944897). **(B)** Representative result of Western blotting (kDa = kilodalton). **(C)** Surface expression levels of tetherin (top) and CD4 (bottom) on cells transfected with Vpu-expressing plasmids. **(D)** The infectivity of released virus was determined by TZM-bl assay. **(E)** The correlation between the level of surface tetherin (shown in panel c, top) and virus infectivity (shown in panel d). The data are expressed as averages and the standard deviations of triplicate assays. In Panels C and D, statistical significance was determined by Student’s *t* test (*P* < 0.05). In Panel E, the correlation between the level of surface tetherin (*x*-axis) and virus infectivity (*y*-axis) was determined by Spearman’s rank test.

Reportedly, the antagonistic effect of Vpu against tetherin is conferred by 3 amino acids in the transmembrane domain of Vpu (^14^AxxxAxxxW^22^ in strain NL4-3). In addition, 2 amino acids in the cytoplasmic domain of Vpu (^52^SxxxS^56^ in strain NL4-3) are essential for down-regulating CD4^42^. To further validate the importance of Vpu on human-to-human viral transmission, we analyzed the *vpu* sequences of transmitted/founder (T/F) viruses, the viruses that are transferred and efficiently established in new individuals^43^,^44^, and recovered from patients at each Fiebig stage (III–V). As shown in Fig. 3A, both of the motifs important for CD4 down-regulation and tetherin antagonism are highly conserved, suggesting that the ability of Vpu to down-regulate tetherin and CD4 is important for establishing HIV-1 infection in new patients.

To demonstrate the link between Vpu activity and establishment of HIV-1 infection, we constructed expression plasmids for the Vpus of T/F viruses and evaluated their ability to infect cell lines. Intriguingly, most of the transfected Vpus down-regulated tetherin but did not efficiently down-regulate CD4 (Fig. 3B–D). Furthermore, the surface expression level of tetherin was significantly negatively correlated with the level of virus release (Fig. 3E; r = −0.678, *P* = 0.0245 by Spearman rank correlation coefficient), suggesting that the ability of Vpu to antagonize tetherin is well correlated with efficient virus release. Collectively, these findings suggest that the potential of Vpu to antagonize tetherin is more important than its anti-CD4 activity, and is well conserved in T/F viruses.

## Discussion

Tetherin suppresses viral release in single-round replication assays in cultured cells such as HeLa cells, and is a recognized inhibitory factor of HIV-1 replication^6^,^7^. However, the anti-viral effect of tetherin seems enigmatic because *vpu*-deficient HIV-1, which cannot counteract tetherin, can replicate in cultures of tetherin-expressing cells such as Jurkat cells and human primary peripheral mononuclear cells (PBMCs). Thus, whether tetherin exerts a lesser effect on HIV-1 replication *in vivo* is a pertinent question. In fact, previous studies by our group and others have demonstrated that *vpu-*deficient HIV-1 efficiently replicates in humanized mouse models^45^,^46^, indicating that Vpu is dispensable for viral propagation *in vivo*. However, Vpu significantly enhances the efficiency of HIV-1 infection in a humanized mouse model^46^. Especially interesting is that only the Vpus of the pandemic virus HIV-1M counteract both tetherin and CD4. The Vpus of the other endemic HIV-1 groups (i.e., N, O, P) antagonize at most one of these protective agents^9^,^10^,^11^. From these observations, we hypothesize that the ability of Vpu to antagonize both tetherin and CD4 increases the infective efficacy of HIV-1M, enabling extensive human-to-human viral transmission. Furthermore, there had been only a dozen of HIV-1N-infected individuals in Cameroon^47^,^48^,^49^, and the antagonistic activity of HIV-1N against tetherin has been shown to be intrinsically weak^11^. Sauter *et al*. revealed that the Vpu of N1Fr2011^50^, a recently designated HIV-1N strain, has acquired anti-tetherin activity after the virus was introduced to Europe from Cameroon^12^. These findings further support a close association between anti-tetherin ability of Vpu and viral epidemics. However, because the efficacy of human-to-human virus transmission cannot be evaluated from experimental studies alone, we supplemented our experimental techniques with a mathematical investigation. Comparing the infectivity of wild-type and *vpu*-deficient HIV-1, we demonstrated that active Vpu confers a 2.38-fold advantage in prevalence of infection (Fig. 2C). This suggests that tetherin plays a “herd immunity” role against HIV-1 in human populations, and that HIV-1M has retaliated by expressing Vpu. Indeed, tetherin impairs the release of a broad spectrum of enveloped viruses^51^. Therefore, the tetherin-mediated herd immunity in human populations may affect the spread of diverse viruses.

Several molecular clones of non-*vpu* encoding HIV-1M strains have been isolated from chronically infected patients. In these strains, designated HXB2^18^, BH8^52^, MAL^53^, and Zr6^54^, the initiation triplet AUG of *vpu* has been deleted^18^,^52^,^53^,^54^ or a frameshift mutation has occurred in the *vpu* ORF^55^. Conversely, all documented T/F viruses that have invaded new individuals encode *vpu*^44^,^56^. Therefore, *vpu* may be essential for infecting new individuals but may be dispensable or less important in viremia maintenance and disease progression. In fact, we have previously revealed that Vpu boosts the number of cell-free viruses and promotes the establishment of viruses in a humanized mouse model^46^. We also found that in earlier pathogenic stages (particularly Fiebig stages III–IV), the motif for tetherin down-regulation (AxxxAxxxW) is conserved and that tetherin is effectively counteracted by the Vpus of T/F viruses (Fig. 3). In contrast, although the motif for CD4 down-regulation (SxxxS) is similarly conserved, CD4 levels may be maintained in the presence of Vpu-producing T/F viruses (Fig. 3). These findings suggest that anti-tetherin activity is more important than CD4 down-regulation, particularly in T/F viruses. However, according to a recent study by Pickering *et al*.^57^, Vpu preserves the ability to downregulate CD4 in infected individuals, and also counteracts tetherin despite the extensive sequence variation of Vpu. They attributed both tetherin antagonism and CD4 down-regulation of strain NL4-3 to at least 4 amino acids, ^19^A, ^23^W, ^53^S, and ^56^S. Therefore, Vpu may facilitate viral propagation during the human-to-human transmission phase.

The experimental-mathematical approach adopted here has quantitatively revealed the replication dynamics of retroviruses^24^,^58^,^59^ and enteroviruses^25^ in cell culture systems. To our knowledge, we present the first estimate of the anti-viral effect of tetherin in human populations by combining experimental and epidemiological data with a structured mathematical model. The data-driven mathematical approach can elucidate viral infection dynamics in ways that are impossible by conventional experimental strategies alone. Especially, the mathematical model incorporates virological information of infected individuals such as the viral load, a crucial parameter for investigating infectious disease spread. As discussed herein, tetherin-mediated herd immunity is revealed only when the set-point viral load is considered as a distribution. If modeled only by the average viral load, the herd immunity effect might be obscured in the population dynamics and the anti-viral effect of tetherin might be underestimated. Thus, quantitative epidemiological details can only be revealed in a multi-scaled modeling approach.

## Additional Information

**How to cite this article**: Iwami, S. *et al*. Pandemic HIV-1 Vpu overcomes intrinsic herd immunity mediated by tetherin. *Sci. Rep*. **5**, 12256; doi: 10.1038/srep12256 (2015).

## Supplementary Material

Supplementary Information

## References

[b1] Worobey M. . Direct evidence of extensive diversity of HIV-1 in Kinshasa by 1960. Nature 455, 661–664, 10.1038/nature07390 (2008). 18833279 PMC3682493

[b2] Gao F. . Origin of HIV-1 in the chimpanzee Pan troglodytes troglodytes. Nature 397, 436–441, 10.1038/17130 (1999). 9989410

[b3] Keele B. F. . Chimpanzee reservoirs of pandemic and nonpandemic HIV-1. Science 313, 523–526, 10.1126/science.1126531 (2006). 16728595 PMC2442710

[b4] Plantier J. C. . A new human immunodeficiency virus derived from gorillas. Nat. Med. 15, 871–872, 10.1038/nm.2016 (2009). 19648927

[b5] Heeney J. L., Dalgleish A. G. & Weiss R. A. Origins of HIV and the evolution of resistance to AIDS. Science 313, 462–466, 10.1126/science.1123016 (2006). 16873637

[b6] Neil S. J., Zang T. & Bieniasz P. D. Tetherin inhibits retrovirus release and is antagonized by HIV-1 Vpu. Nature 451, 425–430 (2008). 18200009 10.1038/nature06553

[b7] Van Damme N. . The interferon-induced protein BST-2 restricts HIV-1 release and is downregulated from the cell surface by the viral Vpu protein. Cell Host Microbe 3, 245–252 (2008). 18342597 10.1016/j.chom.2008.03.001 PMC2474773

[b8] Willey R. L., Maldarelli F., Martin M. A. & Strebel K. Human immunodeficiency virus type 1 Vpu protein induces rapid degradation of CD4. J. Virol. 66, 7193–7200 (1992). 1433512 10.1128/jvi.66.12.7193-7200.1992 PMC240416

[b9] Sauter D. . HIV-1 Group P is unable to antagonize human tetherin by Vpu, Env or Nef. Retrovirology 8, 103, 10.1186/1742-4690-8-103 (2011). 22171785 PMC3285029

[b10] Yang S. J., Lopez L. A., Exline C. M., Haworth K. G. & Cannon P. M. Lack of adaptation to human tetherin in HIV-1 group O and P. Retrovirology 8, 78, 10.1186/1742-4690-8-78 (2011). 21955466 PMC3192746

[b11] Sauter D. . Tetherin-driven adaptation of Vpu and Nef function and the evolution of pandemic and nonpandemic HIV-1 strains. Cell Host Microbe 6, 409–421, 10.1016/j.chom.2009.10.004 (2009). 19917496 PMC2779047

[b12] Sauter D. . Human tetherin exerts strong selection pressure on the HIV-1 group N Vpu protein. PLoS Pathog. 8, e1003093, 10.1371/journal.ppat.1003093 (2012). 23308067 PMC3534379

[b13] Kirchhoff F. Immune evasion and counteraction of restriction factors by HIV-1 and other primate lentiviruses. Cell Host Microbe 8, 55–67, 10.1016/j.chom.2010.06.004 (2010). 20638642

[b14] Loomis-Price L. D. . Correlation between humoral responses to human immunodeficiency virus type 1 envelope and disease progression in early-stage infection. J. Infect. Dis. 178, 1306–1316 (1998). 9780250 10.1086/314436

[b15] Musey L. . Cytotoxic-T-cell responses, viral load, and disease progression in early human immunodeficiency virus type 1 infection. N. Engl. J. Med. 337, 1267–1274, 10.1056/NEJM199710303371803 (1997). 9345075

[b16] Goldstein S., Brown C. R., Dehghani H., Lifson J. D. & Hirsch V. M. Intrinsic susceptibility of rhesus macaque peripheral CD4(+) T cells to simian immunodeficiency virus *in vitro* is predictive of *in vivo* viral replication. J. Virol. 74, 9388–9395 (2000). 11000207 10.1128/jvi.74.20.9388-9395.2000 PMC112367

[b17] Carrington M. . HLA and HIV-1: heterozygote advantage and B*35-Cw*04 disadvantage. Science 283, 1748–1752 (1999). 10073943 10.1126/science.283.5408.1748

[b18] Ratner L. . Complete nucleotide sequences of functional clones of the AIDS virus. AIDS Res. Hum. Retroviruses 3, 57–69 (1987). 3040055 10.1089/aid.1987.3.57

[b19] Metzger V. T., Lloyd-Smith J. O. & Weinberger L. S. Autonomous targeting of infectious superspreaders using engineered transmissible therapies. PLoS Comput. Biol. 7, e1002015, 10.1371/journal.pcbi.1002015 (2011). 21483468 PMC3060167

[b20] Hyman J. M., Li J. & Stanley E. A. The differential infectivity and staged progression models for the transmission of HIV. Math. Biosci. 155, 77–109 (1999). 10067074 10.1016/s0025-5564(98)10057-3

[b21] Kobayashi T. . Identification of amino acids in the human tetherin transmembrane domain responsible for HIV-1 Vpu interaction and susceptibility. J. Virol. 85, 932–945, 10.1128/JVI.01668-10 (2011). 21068238 PMC3020002

[b22] Sato K. . Comparative study on the effect of human BST-2/Tetherin on HIV-1 release in cells of various species. Retrovirology 6, 53, 10.1186/1742-4690-6-53 (2009). 19490609 PMC2702332

[b23] Sato K. . Modulation of human immunodeficiency virus type 1 infectivity through incorporation of tetraspanin proteins. J. Virol. 82, 1021–1033, 10.1128/JVI.01044-07 (2008). 17989173 PMC2224585

[b24] Iwami S. . Quantification system for the viral dynamics of a highly pathogenic simian/human immunodeficiency virus based on an *in vitro* experiment and a mathematical model. Retrovirology 9, 18, 10.1186/1742-4690-9-18 (2012). 22364292 PMC3305505

[b25] Fukuhara M. . Quantification of the dynamics of enterovirus 71 infection by experimental-mathematical investigation. J. Virol. 87, 701–705, 10.1128/JVI.01453-12 (2013). 23097444 PMC3536373

[b26] Neil S. J., Sandrin V., Sundquist W. I. & Bieniasz P. D. An interferon-alpha-induced tethering mechanism inhibits HIV-1 and Ebola virus particle release but is counteracted by the HIV-1 Vpu protein. Cell Host Microbe 2, 193–203, 10.1016/j.chom.2007.08.001 (2007). 18005734 PMC3793644

[b27] Schubert U. . The two biological activities of human immunodeficiency virus type 1 Vpu protein involve two separable structural domains. J. Virol. 70, 809–819 (1996). 8551619 10.1128/jvi.70.2.809-819.1996 PMC189883

[b28] Schubert U., Clouse K. A. & Strebel K. Augmentation of virus secretion by the human immunodeficiency virus type 1 Vpu protein is cell type independent and occurs in cultured human primary macrophages and lymphocytes. J. Virol. 69, 7699–7711 (1995). 7494279 10.1128/jvi.69.12.7699-7711.1995 PMC189711

[b29] Schubert U., Bour S., Willey R. L. & Strebel K. Regulation of virus release by the macrophage-tropic human immunodeficiency virus type 1 AD8 isolate is redundant and can be controlled by either Vpu or Env. J. Virol. 73, 887–896 (1999). 9882289 10.1128/jvi.73.2.887-896.1999 PMC103908

[b30] Theodore T. S. . Construction and characterization of a stable full-length macrophage-tropic HIV type 1 molecular clone that directs the production of high titers of progeny virions. AIDS Res. Hum. Retroviruses 12, 191–194 (1996). 8835195 10.1089/aid.1996.12.191

[b31] Schindler M. . Vpu serine 52 dependent counteraction of tetherin is required for HIV-1 replication in macrophages, but not in *ex vivo* human lymphoid tissue. Retrovirology 7, 1, 10.1186/1742-4690-7-1 (2010). 20078884 PMC2823648

[b32] Perelson A. S. & Ribeiro R. M. Modeling the within-host dynamics of HIV infection. BMC Biol. 11, 96, 10.1186/1741-7007-11-96 (2013). 24020860 PMC3765939

[b33] Perelson A. S. Modelling viral and immune system dynamics. Nat. Rev. Immunol. 2, 28–36, 10.1038/nri700 (2002). 11905835

[b34] Fraser C., Hollingsworth T. D., Chapman R., de Wolf F. & Hanage W. P. Variation in HIV-1 set-point viral load: epidemiological analysis and an evolutionary hypothesis. Proc. Natl. Acad. Sci. USA 104, 17441–17446, 10.1073/pnas.0708559104 (2007). 17954909 PMC2077275

[b35] Fideli U. S. . Virologic and immunologic determinants of heterosexual transmission of human immunodeficiency virus type 1 in Africa. AIDS Res. Hum. Retroviruses 17, 901–910, 10.1089/088922201750290023 (2001). 11461676 PMC2748905

[b36] Geskus R. B.. The H. I. V. RNA setpoint theory revisited. Retrovirology 4, 65, 10.1186/1742-4690-4-65 (2007). 17888148 PMC2206052

[b37] Mellors J. W. . Prognosis in HIV-1 infection predicted by the quantity of virus in plasma. Science 272, 1167–1170 (1996). 8638160 10.1126/science.272.5265.1167

[b38] Anderson R. M. & May R. M. Immunisation and herd immunity. Lancet 335, 641–645 (1990). 1969023 10.1016/0140-6736(90)90420-a

[b39] Anderson R. M. & May R. M. Epidemiological parameters of HIV transmission. Nature 333, 514–519, 10.1038/333514a0 (1988). 3374601

[b40] Velasco-Hernandez J. X., Gershengorn H. B. & Blower S. M. Could widespread use of combination antiretroviral therapy eradicate HIV epidemics? Lancet Infect. Dis. 2, 487–493 (2002). 12150848 10.1016/s1473-3099(02)00346-8

[b41] Rashid H., Khandaker G. & Booy R. Vaccination and herd immunity: what more do we know? Curr. Opin. Infect. Dis. 25, 243–249, 10.1097/QCO.0b013e328352f727 (2012). 22561998

[b42] Vigan R. & Neil S. J. Determinants of tetherin antagonism in the transmembrane domain of the human immunodeficiency virus type 1 Vpu protein. J. Virol. 84, 12958–12970 (2010). 20926557 10.1128/JVI.01699-10 PMC3004320

[b43] Keele B. F. . Identification and characterization of transmitted and early founder virus envelopes in primary HIV-1 infection. Proc. Natl. Acad. Sci. USA 105, 7552–7557, 10.1073/pnas.0802203105 (2008). 18490657 PMC2387184

[b44] Salazar-Gonzalez J. F. . Genetic identity, biological phenotype, and evolutionary pathways of transmitted/founder viruses in acute and early HIV-1 infection. J. Exp. Med. 206, 1273–1289, 10.1084/jem.20090378 (2009). 19487424 PMC2715054

[b45] Dave V. P., Hajjar F., Dieng M. M., Haddad E. & Cohen E. A. Efficient BST2 antagonism by Vpu is critical for early HIV-1 dissemination in humanized mice. Retrovirology 10, 128, 10.1186/1742-4690-10-128 (2013). 24195843 PMC4226203

[b46] Sato K. . Vpu augments the initial burst phase of HIV-1 propagation and downregulates BST2 and CD4 in humanized mice. J. Virol. 86, 5000–5013, 10.1128/JVI.07062-11 (2012). 22357275 PMC3347374

[b47] Simon F. . Identification of a new human immunodeficiency virus type 1 distinct from group M and group O. Nat. Med. 4, 1032–1037, 10.1038/2017 (1998). 9734396

[b48] Vallari A. . Four new HIV-1 group N isolates from Cameroon: Prevalence continues to be low. AIDS Res. Hum. Retroviruses 26, 109–115, 10.1089/aid.2009.0178 (2010). 20059396

[b49] Yamaguchi J. . Identification of HIV type 1 group N infections in a husband and wife in Cameroon: viral genome sequences provide evidence for horizontal transmission. AIDS Res. Hum. Retroviruses 22, 83–92, 10.1089/aid.2006.22.83 (2006). 16438650

[b50] Delaugerre C., De Oliveira F., Lascoux-Combe C., Plantier J. C. & Simon F. HIV-1 group N: travelling beyond Cameroon. Lancet 378, 1894, 10.1016/S0140-6736(11)61457-8 (2011). 22118443

[b51] Sato K., Gee P. & Koyanagi Y. Vpu and BST2: Still Not There Yet? Front. Microbiol. 3, 131, 10.3389/fmicb.2012.00131 (2012). 22509177 PMC3321438

[b52] Ratner L. . Complete nucleotide sequence of the AIDS virus, HTLV-III. Nature 313, 277–284 (1985). 2578615 10.1038/313277a0

[b53] Alizon M., Wain-Hobson S., Montagnier L. & Sonigo P. Genetic variability of the AIDS virus: nucleotide sequence analysis of two isolates from African patients. Cell 46, 63–74 (1986). 2424612 10.1016/0092-8674(86)90860-3

[b54] Srinivasan A. . Molecular characterization of human immunodeficiency virus from Zaire: nucleotide sequence analysis identifies conserved and variable domains in the envelope gene. Gene 52, 71–82 (1987). 3036660 10.1016/0378-1119(87)90396-9

[b55] Sanchez-Pescador R. . Nucleotide sequence and expression of an AIDS-associated retrovirus (ARV-2). Science 227, 484–492 (1985). 2578227 10.1126/science.2578227

[b56] Parrish N. F. . Phenotypic properties of transmitted founder HIV-1. Proc. Natl. Acad. Sci. U. S. A. 110, 6626–6633, 10.1073/pnas.1304288110 (2013). 23542380 PMC3637789

[b57] Pickering S. . Preservation of tetherin and CD4 counter-activities in circulating Vpu alleles despite extensive sequence variation within HIV-1 infected individuals. PLoS Pathog. 10, e1003895, 10.1371/journal.ppat.1003895 (2014). 24465210 PMC3900648

[b58] Iwami S., Koizumi Y., Ikeda H. & Kakizoe Y. Quantification of viral infection dynamics in animal experiments. Front. Microbiol. 4, 264, 10.3389/fmicb.2013.00264 (2013). 24058361 PMC3767920

[b59] Kobayashi T. . Quantification of deaminase activity-dependent and -independent restriction of HIV-1 replication mediated by APOBEC3F and APOBEC3G through experimental-mathematical investigation. J. Virol. 88, 5881–5887, 10.1128/JVI.00062-14 (2014). 24623435 PMC4019142

