# Supplementary material for: Pandemic HIV-1 Vpu overcomes intrinsic herd immunity mediated by tetherin
Source: Sci Rep. 2015 Jul 17;5:12256. doi: 10.1038/srep12256 (PMC4505337; doi:10.1038/srep12256)
Supplement: Supplementary Information [file srep12256-s1.pdf]

Pandemic HIV-1 Vpu overcomes intrinsic herd immunity mediated by tetherin: Supporting Informations

・SI Texts

・SI References

・SI Figures

・SI Tables

Shingo Iwami[1,2,3]*†, Kei Sato[4,3]*†, Satoru Morita[5,3], Hisashi Inaba[6,3], Tomoko Kobayashi[7], Junko S. Takeuchi[4], Yuichi Kimura[4], Naoko Misawa[4], Fengrong Ren[8], Yoh Iwasa[1], Kazuyuki Aihara[9,10] & Yoshio Koyanagi[4]

[1]Mathematical Biology Laboratory, Department of Biology, Faculty of Sciences, Kyushu University, Fukuoka, Fukuoka 8128581, Japan. [2]PRESTO, JST, Kawaguchi, Saitama 3320012, Japan. [3]CREST, JST, Kawaguchi, Saitama 3320012, Japan. [4]Laboratory of Viral Pathogenesis, Institute for Virus Research, Kyoto University, Kyoto, Kyoto 6068507, Japan. [5]Department of Mathematical and Systems Engineering, Shizuoka University, Hamamatsu, Shizuoka 4328561, Japan. [6]Graduate School of Mathematical Sciences, The University of Tokyo, Meguro-ku, Tokyo 1538914, Japan. [7]Laboratory for Animal Health, Department of Animal Science, Faculty of Agriculture, Tokyo University of Agriculture, Atsugi, Kanagawa 2430034, Japan. [8]Department of Bioinformatics, Medical Research Institute, Tokyo Medical and Dental University, Tokyo 1138510, Japan. [9]Institute of Industrial Science, The University of Tokyo, Meguro-ku, Tokyo 1538505, Japan. [10]Graduate School of Information Science and Technology, The University of Tokyo, Meguro-ku, Tokyo 1138656, Japan.

† These authors contributed equally to this study.

* Correspondence and requests for materials should be addressed to S.I. (email: siwami@kyushu-u.org) or K.S. (email: ksato@virus.kyoto-u.ac.jp).

Text S1: Quantifying virus production rate of wild-type and *vpu*-deficient HIV-1

To quantify the Vpu function, we infected cell cultures with wild-type and *vpu*-deficient HIV-1 as previously described [1,2]. Briefly, cultures of PHA-activated human PBMCs were infected with wild-type (or *vpu*-deficient) HIV-1 and sampled at 3-day intervals over 39 consecutive days. Each day, most of the virus (85.4%) was removed from the culture supernatant for viral load assay (p24/ml), and fresh medium was added. The experiment was performed in triplicate. From the results, we obtained the parameters of the following basic mathematical model of the viral infection dynamics [3];

$$\frac{dT(t)}{dt} = -\omega T(t)V(t), \qquad \frac{dI(t)}{dt} = \omega T(t)V(t) - \delta I(t), \qquad \frac{dV(t)}{dt} = pI(t) - cV(t), \quad (\mathrm{S1})$$

where $T(t)$ and $I(t)$ are the numbers of target (susceptible) cells, and infected (virus-producing) cells respectively per ml of medium, and $V(t)$ is the amount of viral protein p24 per ml of medium. The parameters δ, c, ω and p represent the death rate of infected cells, the clearance rate of virions, the rate constant for viral infection of target cells, and the viral reproduction rate of an infected cell, respectively. Because tetherin impairs viral release by tethering bud virions to the surfaces of HIV-1-producing cells, the physical virion retention by tetherin should be reflected in the virus production rate, p. Here we assumed that wild-type HIV-1 is produced at a higher rate than *vpu*-deficient virus because Vpu enhances the release of HIV-1 virions by antagonizing tetherin. The other parameters are assumed identical in wild-type and mutant virus.

To quantitatively investigate the role of Vpu (i.e., to determine how the viral dynamics differ between wild-type and *vpu*-deficient HIV-1), we simultaneously fitted the above equations to the time-course data of the viral load by nonlinear least-squares regression (using the *FindMinimum* package of *Mathematica 9.0*). This technique minimizes the sum of squared residuals (SSR). The fitted parameter values are listed in **Table S1** and the model behavior using these best-fit parameter estimates is presented together with the data in **Fig. S1**. Furthermore, we analyzed

an additional 15 datasets presented in previous studies [4-10] and estimated their parameter values (**Table S1**). The average proportional production rate of wild-type HIV-1 to *vpu*-deficient HIV-1 was then estimated as

$$p_{WT}/p_{MT} = 1.54. \quad (S2)$$

This implies that tetherin effectively suppresses viral production in HIV-1 infected cells.

Text S2: Set-point viral load of *vpu*-deficient HIV-1 infected patients

To calculate the set-point viral load *in vivo*, we added the normal turnover dynamics of target cells (i.e., supply (λ) and death ($uT(t)$) of target cells) into Eqs. (S1) [11,12]:

$$\frac{dT(t)}{dt} = \lambda - uT(t) - \omega T(t)V(t), \qquad \frac{dI(t)}{dt} = \omega T(t)V(t) - \delta I(t), \qquad \frac{dV(t)}{dt} = pI(t) - cV(t). \quad (S3)$$

Here, $T(t)$ and $I(t)$ are the numbers of target and infected cells, respectively, per ml of peripheral blood (PB), and $V(t)$ is the number of viral RNA copies per ml of PB. From the internal equilibrium relationship, the set-point viral load is given by

$$V = \frac{\lambda p}{\delta c} - \frac{u}{\omega}, \qquad (S4)$$

if $\lambda\omega p/u\delta c \geq 1$; otherwise $V = 0$.

Hereafter, we investigate the impact of Vpu on the set-point viral load, assuming biologically plausible parameter values. If 1 ml of PB contains 1.0×10^6 CD4 T cells, and 1% of the CD4 T cells are susceptible to HIV-1 infection, the density of susceptible CD4 T cells is $(1.0 \times 10^4/\mathrm{ml})$ [13]. Because the average life span of CD4 T cells is around 100 days, we set $u = 0.01$ (day^{-1}) [14], and thereby calculate the supply rate constant of the susceptible CD4 T cells as $\lambda = 100$ (cells/ml). The death rate of infected cells and the viral clearance rate have been well estimated from treatment data collected during the chronic phase of HIV-1 infected patients, and are here assumed as $\delta = 0.65$ (day^{-1}) [15,16] and $c = 30$ (day^{-1}) [17,18], respectively. Furthermore, because the daily virus production rate *in vivo* is around 4.0×10^4 RNA copies/ml per day [13,19], we take $p_{WT} = 3.1 \times 10^4$ (RNA copies/ml・day^{-1}). Note that a small change of the value of p_{WT} do not affect our conclusion (see **Text S6**). Finally, to satisfy the typical set-point value of HIV-1M infected patients, namely $V_{WT} = 1.0 \times 10^5$ copies/ml [20,21], we determine the infection rate as $\omega = 1.69 \times 10^{-7}$ (RNA copies/ml・day)$^{-1}$ from Eq. (S4). Therefore, if *vpu* is absent in HIV-1M, the viral production rate reduces to $p_{MT} = p_{WT}/1.54 = 2.01 \times 10^3$ copies/ml・day^{-1}, and the set-point becomes $V_{MT} = 4.4 \times 10^4$ copies/ml.

This implies that Vpu significantly enhances the set-point viral load. From Eqs. (S2) and (S4), we remark that V_{MT} and V_{WT} are related through the composite parameter u/ω:

$$V_{MT} = \frac{V_{WT}}{1.54} - 0.351\frac{u}{\omega}, \qquad (S5)$$

if $V_{WT} \geq 0.54u/\omega$ and $V_{MT} = 0$ otherwise. The sensitivity of the composite parameter (u/ω) is discussed in **Text S6**.

Text S3: Derivation of the basic reproduction number

Here we outline a method for calculating the basic reproduction number in HIV infection. In our mathematical model described in **Methods**, $S(t,V)$ represents the number of susceptible individuals who will acquire a set-point viral load V if infected by HIV-1 in the future, and $I(t,V)$ is the number of infected individuals with set-point viral load V at time t. We assume that this host heterogeneity is independent of time and infection status.

Clearly, the disease-free steady state of the model is given by

$$\left(S_0(V), I_0(V)\right) = \left(\frac{b(V)}{d}, 0\right).$$

Let $N_0 = \int_0^\infty S_0(V)dV$ be the host population size in the disease-free steady state. Then the linearized equation at the disease-free steady state is given by

$$\frac{\partial I(t,V)}{\partial t} = \frac{S_0(V)}{N_0}\int_0^\infty c\beta(W)I(t,W)dW - \mu(V)I(t,V).$$

Let

$$B(t,V) := \frac{S_0(V)}{N_0}\int_0^\infty c\beta(W)I(t,W)dW$$

be the density of newly infected individuals at time t. From the variation of constants formula, the vector-valued renewal equation is given by

$$B(t,V) = \frac{S_0(V)}{N_0}\int_0^\infty c\beta(W)\left[e^{-\mu(W)t}I(0,W) + \int_0^t e^{-\mu(W)(t-s)}B(s,W)ds\right]dW$$
$$= G(t,V) + \int_0^t \left(\Psi(s)B(t-s,\cdot)\right)(V)ds\,,$$

where

$$G(t,V) \coloneqq \frac{S_0(V)}{N_0}\int_0^\infty c\beta(W)e^{-\mu(W)t}I(0,W)dW,$$

denotes the initial data and the integral kernel Ψ is a positive operator on L^1 defined by

$$(\Psi(s)f)(V) \coloneqq \frac{S_0(V)}{N_0}\int_0^\infty e^{-\mu(W)s} c\beta(W) f(W) dW, \qquad f \in L^1(0,\infty).$$

Therefore we can define the next generation operator K as follows [22,23]:

$$(Kf)(V) \coloneqq \int_0^\infty (\Psi(s)f)(V) ds = \frac{S_0(V)}{N_0}\int_0^\infty \frac{c\beta(W)}{\mu(W)} f(W) dW, \qquad f \in L^1(0,\infty).$$

Because the basic reproduction number is the positive eigenvalue of K, solving the eigenvalue problem $Kf = R_0 f$, we obtain

$$R_0 = \int_0^\infty \frac{c\beta(W)}{\mu(W)} \frac{S_0(W)}{N_0} dW$$

where S_0 is the positive eigenvector of K associated with R_0.

Let $\rho(V) = S_0(V)/N_0$ be the trait (normalized) distribution of the host population in the disease-free steady state. Then we have

$$R_0 = \int_0^\infty \frac{c\beta(V)}{\mu(V)} \rho(V) dV. \quad (S6)$$

Text S4: Calculation of the basic reproduction number in wild-type HIV-1M infected patients.

The basic reproduction number was estimated from the distribution of the viral load in the Zambian Transmission Study. In previously published papers [20,21], the transmission probability and the mean duration of the asymptomatic phase were given as

$$\beta(V) = \beta_{max} V^{k_\beta} / \left(V^{k_\beta} + {V_{\beta 50}}^{k_\beta}\right),$$
$$1/\mu(V) = D_{max} {D_{50}}^{k_D} / \left(V^{k_D} + {V_{D50}}^{k_D}\right),$$

with estimated parameter values $\beta_{max} = 0.317$, $v_{\beta 50} = 13938$, $k_\beta = 1.08$, $D_{max} = 25.4$, $v_{D50} = 3058$ and $k_D = 0.41$. Therefore, to numerically determine R_0, we need to estimate $\rho(V) = S_0(V)/N_0 = b(V)/(d \times N_0)$ in Eq. (S6).

Let us examine the relationship between $\rho(V) = S_0(V)/N_0$ and the observed distribution of set-point viral load. Suppose that the host population exists in an endemic steady state. Let $S^*(V)$ and $I^*(V)$ be the density of susceptible and asymptomatically infected individuals, respectively, in this state. Then we have

$$b(V) - dS^*(V) - \frac{S^*(V)}{N^*} \langle c\beta, I^* \rangle = 0,$$
$$\frac{S^*(V)}{N^*} \langle c\beta, I^* \rangle - \mu(V) I^*(V) = 0,$$

where N^* denotes the host population size in endemic steady state and

$$\langle c\beta, I^* \rangle := \int_0^\infty c\beta(W) I^*(W) dW.$$

It easily shown that

$$I^*(V) = \frac{S^*(V)\langle c\beta, I^* \rangle}{N^* \mu(V)} = S_0(V) \frac{\langle c\beta, I^* \rangle}{N^* \mu(V) + (\mu(V)/d)\langle c\beta, I^* \rangle}.$$

From the endemic steady state relationship, we obtain

$$I^*(V) = \frac{b(V)}{\mu(V)} \frac{\langle c\beta, I^* \rangle}{dN^* + \langle c\beta, I^* \rangle}.$$

Assuming a skew-lognormal distribution $b(V)$:

$$b(V) = dN(0)\frac{\exp\left(-\frac{(\log V-\zeta)^2}{2\sigma^2}\mathrm{Erfc}\left[-\frac{\alpha(\log V-\zeta)}{\sqrt{2}\sigma}\right]\right)}{\sqrt{2\pi}\sigma} \quad (S7),$$

we find that the distribution of the logarithm of the viral load in the Zambian Transmission Study is well described by the above proportionality relationship $I^*(V) \propto b(V)/\mu(V)$ (i.e., by the weighted skew-normal distribution). Hence, we used the skew-normal distribution and estimated the parameters ζ, σ and α by maximum likelihood (using the *FindMaximum* package of *Mathematica 9.0*), assuming that HIV-1M transmission in the Zambian Transmission Study has already reached steady-state. The estimated parameters $\zeta = 5.87$, $\sigma = 1.07$, and $\alpha = -3.6$, and the probability density function $b(V)$ is shown in **Fig. S2**. Assuming previously-derived parameters $d = 1/35$ years (corresponding to adults aged 15–49 years) and $c = 4.02$ partners/year [20,21], the basic reproduction number of HIV-1M is calculated as $R_0^{WT} = 4.67$.

Text S5: Predicted distribution of set-point viral load and the basic reproduction number of *vpu*-deficient HIV-1 infected patients

We then investigated how Vpu of HIV-1M contributes to the shape of the distribution of the set-point viral load. In other words, we calculated the distribution of set-point viral load under conditions of lost Vpu function (i.e., *vpu*-deficient HIV-1). Interestingly, if the set-point viral load is below $V_{WT} = 3.2 \times 10^4$ copies/ml, the *vpu*-deficient HIV-1 cannot establish infection (i.e., $V_{MT} = 0$) because tetherin exerts a powerful anti-HIV effect (see above). This implies that, if the HIV-1M in the Zambian Transmission Study loses its Vpu function, approximately 20% of infected patients with lower viral load would not become infected by *vpu*-deficient HIV-1 (Fig. 2a). Hence, the anti-HIV effect of tetherin potentially drives intrinsic herd immunity in HIV transmission, provided that tetherin is not antagonized. Assuming that the birth rate $b(V)$ obeys Eq. (S7), and the relationship between V_{WT} and V_{MT} is given by Eq. (S5), we obtained the distribution of V_{MT}. By the method of **Text S3**, we then calculated the basic reproduction number of *vpu*-deficient HIV-1 (R_0^{MT}).

Text S6: Sensitivity of HIV-1 M infection parameters to tetherin-mediated herd immunity

As mentioned in **Text S2**, V_{MT} and V_{WT} are related through the composite parameter u/ω. In Eq. (S8), ε is defined as a "control parameter":

$$V_{MT} = \frac{V_{WT}}{1.54} - 0.351\left(\frac{u}{\omega} \times \varepsilon\right). \qquad (S8)$$

Fig. S3 shows the sensitivity of the composite parameter on (a) the predicted set-point viral load of *vpu*-deficient HIV-1, (b) the percentage of the population protected by tetherin, (c) the ratio of increases in basic reproduction numbers of wild-type and mutant virus (i.e., R_0^{WT}/R_0^{MT}), and (d) the ratio of increases in viral prevalence of wild-type and mutant virus. In these analyses, ε was varied from 50% to 200% (i.e., $\varepsilon \in [0.5, 2.0]$), while maintaining $\lambda p/\delta c - \varepsilon u/\omega = 1.0 \times 10^5$. Although the results of (a–d) depend on ε, we impose a biologically plausible perturbation on u/ω; that is, we preserve the tetherin-mediated herd immunity in (d).

SI References

1. Fukuhara, M. *et al.* Quantification of the dynamics of enterovirus 71 infection by experimental-mathematical investigation. *J. Virol.* **87**, 701-705, doi:10.1128/JVI.01453-12 (2013).
2. Iwami, S. *et al.* Quantification system for the viral dynamics of a highly pathogenic simian/human immunodeficiency virus based on an in vitro experiment and a mathematical model. *Retrovirology* **9**, 18, doi:10.1186/1742-4690-9-18 (2012).
3. Iwami, S., Koizumi, Y., Ikeda, H. & Kakizoe, Y. Quantification of viral infection dynamics in animal experiments. *Frontiers in microbiology* **4**, 264, doi:10.3389/fmicb.2013.00264 (2013).
4. Van Damme, N. *et al.* The interferon-induced protein BST-2 restricts HIV-1 release and is downregulated from the cell surface by the viral Vpu protein. *Cell Host Microbe* **3**, 245-252 (2008).
5. Neil, S. J., Sandrin, V., Sundquist, W. I. & Bieniasz, P. D. An interferon-alpha-induced tethering mechanism inhibits HIV-1 and Ebola virus particle release but is counteracted by the HIV-1 Vpu protein. *Cell Host Microbe* **2**, 193-203, doi:10.1016/j.chom.2007.08.001 (2007).
6. Schubert, U. *et al.* The two biological activities of human immunodeficiency virus type 1 Vpu protein involve two separable structural domains. *J. Virol.* **70**, 809-819 (1996).
7. Schubert, U., Clouse, K. A. & Strebel, K. Augmentation of virus secretion by the human immunodeficiency virus type 1 Vpu protein is cell type independent and occurs in cultured human primary macrophages and lymphocytes. *J. Virol.* **69**, 7699-7711 (1995).
8. Schubert, U., Bour, S., Willey, R. L. & Strebel, K. Regulation of virus release by the macrophage-tropic human immunodeficiency virus type 1 AD8 isolate is redundant and can be controlled by either Vpu or Env. *J. Virol.* **73**, 887-896 (1999).
9. Theodore, T. S. *et al.* Construction and characterization of a stable full-length macrophage-tropic HIV type 1 molecular clone that directs the production of high titers of progeny virions. *AIDS Res. Hum. Retroviruses* **12**, 191-194 (1996).
10. Schindler, M. *et al.* Vpu serine 52 dependent counteraction of tetherin is required for HIV-1 replication in macrophages, but not in ex vivo human lymphoid tissue. *Retrovirology* **7**, 1, doi:10.1186/1742-4690-7-1 (2010).
11. Perelson, A. S. & Ribeiro, R. M. Modeling the within-host dynamics of HIV infection. *BMC Biol.* **11**, 96, doi:10.1186/1741-7007-11-96 (2013).
12. Ratner, L. *et al.* Complete nucleotide sequence of the AIDS virus, HTLV-III. *Nature* **313**, 277-284 (1985).
13. Stafford, M. A. *et al.* Modeling plasma virus concentration during primary HIV infection. *Journal of theoretical biology* **203**, 285-301, doi:10.1006/jtbi.2000.1076 (2000).

14 De Boer, R. J. & Perelson, A. S. Quantifying T lymphocyte turnover. *J. Theor. Biol.* **327**, 45-87, doi:10.1016/j.jtbi.2012.12.025 (2013).

15 Markowitz, M. *et al.* A novel antiviral intervention results in more accurate assessment of human immunodeficiency virus type 1 replication dynamics and T-cell decay in vivo. *J. Virol.* **77**, 5037-5038 (2003).

16 Perelson, A. S. *et al.* Decay characteristics of HIV-1-infected compartments during combination therapy. *Nature* **387**, 188-191, doi:10.1038/387188a0 (1997).

17 Perelson, A. S., Neumann, A. U., Markowitz, M., Leonard, J. M. & Ho, D. D. HIV-1 dynamics in vivo: virion clearance rate, infected cell life-span, and viral generation time. *Science* **271**, 1582-1586 (1996).

18 Ramratnam, B. *et al.* Rapid production and clearance of HIV-1 and hepatitis C virus assessed by large volume plasma apheresis. *Lancet* **354**, 1782-1785, doi:10.1016/S0140-6736(99)02035-8 (1999).

19 Chen, H. Y., Di Mascio, M., Perelson, A. S., Ho, D. D. & Zhang, L. Determination of virus burst size in vivo using a single-cycle SIV in rhesus macaques. *Proc. Natl. Acad. Sci. U. S. A.* **104**, 19079-19084, doi:10.1073/pnas.0707449104 (2007).

20 Fraser, C., Hollingsworth, T. D., Chapman, R., de Wolf, F. & Hanage, W. P. Variation in HIV-1 set-point viral load: epidemiological analysis and an evolutionary hypothesis. *Proc. Natl. Acad. Sci. U. S. A.* **104**, 17441-17446, doi:10.1073/pnas.0708559104 (2007).

21 Metzger, V. T., Lloyd-Smith, J. O. & Weinberger, L. S. Autonomous targeting of infectious superspreaders using engineered transmissible therapies. *PLoS Comput. Biol.* **7**, e1002015, doi:10.1371/journal.pcbi.1002015 (2011).

22 Inaba, H. On a new perspective of the basic reproduction number in heterogeneous environments. *J. Math. Biol.* **65**, 309-348, doi:10.1007/s00285-011-0463-z (2012).

23 Diekmann, O., Heesterbeek, J. A. P. & Britton, T. *Mathematical Tools for Understanding Infectious Disease Dynamics*. (Princeton University Press, 2013).

SI Figures

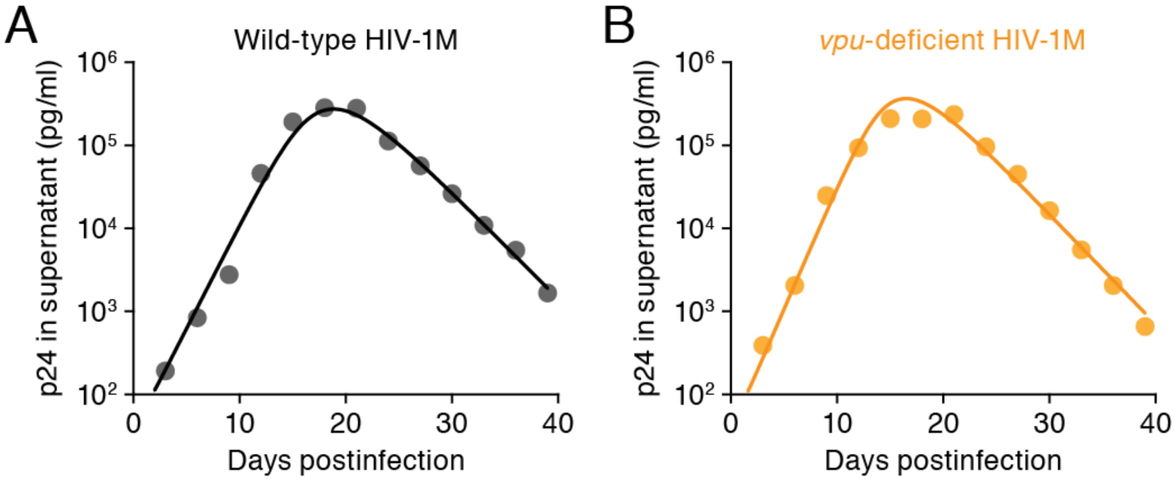

Figure S1 | Dynamics of wild-type and *vpu*-deficient HIV-1 in PBMC. The average number of viral load (pg/ml/culture) in wild-type HIV-1 (a) and *vpu*-deficient HIV-1 (b). The symbols and error bars denote the averages and their standard deviations, respectively. The continuous line depicts the best fit of the model to the average data obtained from triplicate experiments ($\omega = 1.99 \times 10^{-6}$, $p_{WT} = 0.328$, $p_{MT} = 0.270$, $\delta = 0.310$, $c = 0.64$).

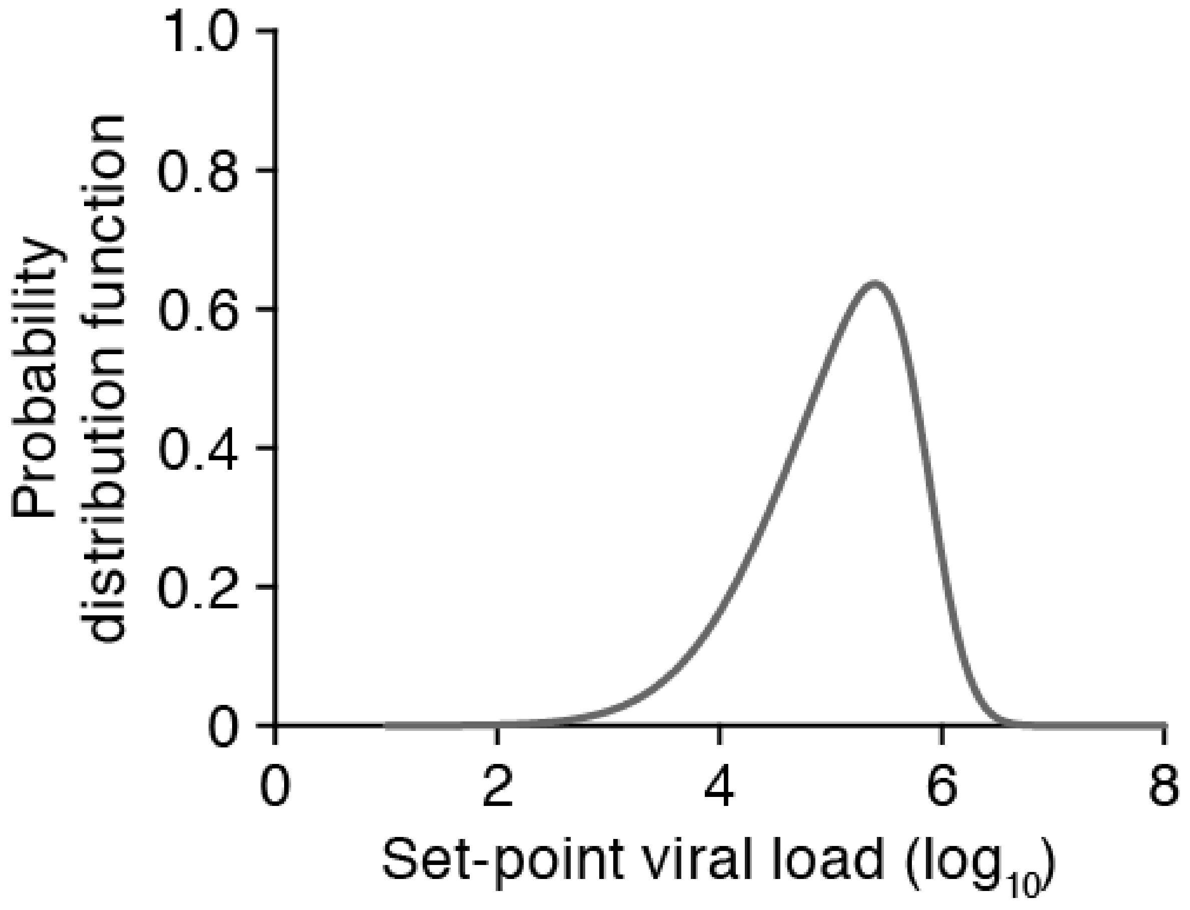

Figure S2 | Set-point viral load and probability density function of birth rate. The probability density function of the birth rate is estimated from the distribution of the viral load in the Zambian Transmission Study, assuming a skew-lognormal distribution of birth rate. The estimated birth rate parameters are $\zeta = 5.87$, $\sigma = 1.07$, and $\alpha = -3.6$.

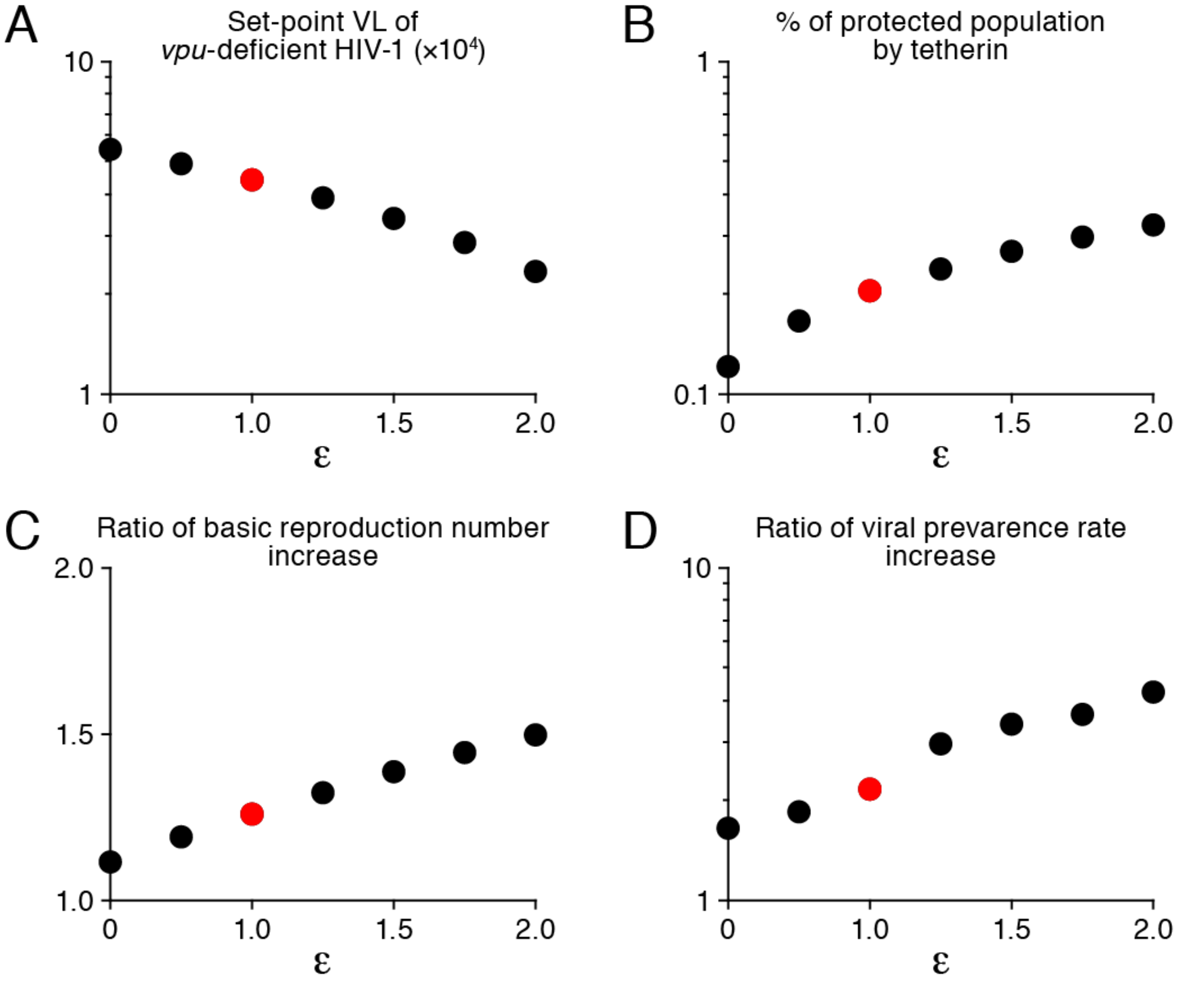

Figure S3 | Sensitivity of composed parameter. (a) the predicted set-point viral load of *vpu-deficiency* HIV-1, (b) the percentage of the population protected by tetherin, (c) the ratio of basic reproduction number increases (i.e., R_0^{WT}/R_0^{MT}), and (d) the ratio of viral prevalence increases, as functions of ε (where ε is varied from 50% to 200%).

SI Tables

Table S1 | Estimated parameter values of viral infection dynamics of wild-type and *vpu*-deficient HIV-1

Virus (unit)	Cells†	Estimated parameters						Source
		ω	p_{WT}	p_{MT}	p_{WT}/p_{MT}	δ	c *1	
HIV-1 NL4-3 (p24)	PBMC	$(1.97\pm0.06)\times 10^{-6}$	0.33±0.004	0.27±0.009	1.22	0.31±0.015	0.64	This study
HIV-1 NL4-3 (Virus yield (RLU))	PBMC	4.48×10^{-5}	0.96	0.57	1.68	1.50	0.96	Fig. 6B in reference[5]
HIV-1 NL(AD8) (RT activity)	PBMC	4.68×10^{-5}	0.0061	0.0036	1.69	0.18	0.96	Fig. 4A in reference[7]
HIV-1 AD8 (RT activity)	PBMC	1.78×10^{-5}	0.028	0.017	1.65	0.20	0.96	Fig. 4B in reference[7]
HIV-1 NL4-3 (RT activity)	PBMC	5.00×10^{-7}	1.61	0.83	1.94	0.56	1.33	Fig. 1A in reference[6]
HIV-1 AD8 (RT activity)	PBMC	2.09×10^{-8}	12.0	10.0	1.20	0.24	0.96	Fig. 2C in reference[8]
HIV-1 NL4-3 (Virus yield (RLU))	Jurkat	5.52×10^{-5}	1.88	1.72	1.09	3.30	0.96	Fig. 6A in reference[5]
HIV-1 NL4-3 (RT activity)	A3.01	3.47×10^{-8}	17.4	15.8	1.10	1.91	1.33	Fig. 1B in reference[6]
HIV-1 NL4-3 (p24)	CEM	—	—	—	1.19 *2	—	—	Fig. 1D in reference[4]
HIV-1 AD8 (RT activity)	MDM	6.14×10^{-5}	0.043	0.029	1.48	0.055	0.96	Fig. 3C in reference[9]
HIV-1 NL4-3 (RT activity)	MDM	5.88×10^{-5}	0.025	0.0035	7.14 *3	0.038	0.64	Fig. 4A in reference[7]

HIV-1 AD8 (RT activity)	MDM	2.36×10^{-5}	0.038	0.016	2.38	0.054	0.64	Fig. 4C in reference[7]
HIV-1 AD8 (RT activity)	MDM	2.01×10^{-10}	1840.8	979.6	1.88	0.054	0.64	Fig. 2B in reference[8]
HIV-1 NL4-3 (X4) (p24)	HLT	—	—	—	1.95 *2	—	—	Fig. 4A in reference[10]
HIV-1 NL4-3 (R5) (p24)	HLT	—	—	—	1.13 *2	—	—	Fig. 4A in reference[10]

† PBMC, peripheral mononuclear cell; MDM, monocyte-derived macrophage; HLT, human lymphoid tissue.

*1 The clearance rate of virus was estimated based on the frequency of medium replacement.

*2 Only p_{WT}/p_{MT} was estimated because of sparse experimental data.

*3 The outlier was removed from the calculation of the average in the **Result**.

Table S2 | Parameter values used in simulations of the structured epidemiological model

Symbol	Description	Unit	Value	Source
ζ	Position parameter of skew-lognormal distribution for $b(V)$	---	5.87	Estimated in **Text S4**
σ	Variability parameter of skew-lognormal distribution for $b(V)$	---	1.07	Estimated in **Text S4**
α	Skewness parameter of skew-lognormal distribution for $b(V)$	---	-3.6	Estimated in **Text S4**
d	Rate at which individuals leave the model population (i.e., removal rate)	per year	$1/35$	Adult age 15-49 years
β_{max}	Maximum transmission probability	per year	0.317	Estimated in reference [20]
$v_{\beta_{50}}$	Viral load at which the transmission probability is half of β_{max}	RNA copies/ml	13938	Estimated in reference [20]
k_β	Steepness of $\beta(V)$ as a function of set-point viral load	---	1.08	Estimated in reference [20]
c	Annual partner change rate	per year	4.02	Estimated in reference [20,21]
D_{max}	Maximum duration of the asymptomatic period	years	25.4	Estimated in reference [20]
$v_{D_{50}}$	Viral load at which the duration of the asymptomatic period is half of D_{max}	RNA copies/ml	3058	Estimated in reference [20]
k_D	Steepness of $\mu(V)$ as a function of set-point viral load	---	0.41	Estimated in reference [20]
$N(0)$	Initial population size of total individuals	individuals	10^6	An arbitrary number
$I(0,V)$	Initial population size of infected individuals	individuals	1	An arbitrary number
